# Single-cell RNA sequencing analysis reveals the heterogeneity of IL-10 producing regulatory B cells in lupus-prone mice

**DOI:** 10.3389/fimmu.2023.1282770

**Published:** 2023-12-14

**Authors:** Andrea R. Daamen, Razan M. Alajoleen, Amrie C. Grammer, Xin M. Luo, Peter E. Lipsky

**Affiliations:** ^1^ AMPEL BioSolutions LLC and the RILITE Research Institute, Charlottesville, VA, United States; ^2^ Department of Biomedical Sciences and Pathology, Virginia-Maryland College of Veterinary Medicine, Virginia Tech, Blacksburg, VA, United States

**Keywords:** lupus, Breg, single-cell, transcriptomics, bioinformatics

## Abstract

**Introduction:**

B cells can have both pathogenic and protective roles in autoimmune diseases, including systemic lupus erythematosus (SLE). Deficiencies in the number or immunosuppressive function of IL-10 producing regulatory B cells (Bregs) can cause exacerbated autoimmune inflammation. However, the exact role of Bregs in lupus pathogenesis has not been elucidated.

**Methods:**

We carried out gene expression analysis by scRNA-seq to characterize differences in splenic Breg subsets and molecular profiles through stages of disease progression in lupus-prone mice. Transcriptome-based changes in Bregs from mice with active disease were confirmed by phenotypic analysis.

**Results:**

We found that a loss of marginal zone (MZ) lineage Bregs, an increase in plasmablast/plasma cell (PB-PC) lineage Bregs, and overall increases in inflammatory gene signatures were characteristic of active disease as compared to Bregs from the pre-disease stage. However, the frequencies of both MZ Bregs and PB-PCs expressing IL-10 were significantly decreased in active-disease mice.

**Conclusion:**

Overall, we have identified changes to the repertoire and transcriptional landscape of Breg subsets associated with active disease that provide insights into the role of Bregs in lupus pathogenesis. These results could inform the design of Breg-targeted therapies and interventions to restore Breg suppressive function in autoimmunity.

## Introduction

B cells are typically thought of as positive effectors of autoimmunity through the production of autoantibodies and inflammatory cytokines. However, subsets of B cells generally characterized by the production of interleukin-10 (IL-10) and broadly labeled regulatory B cells (Bregs) can also function as negative regulators of the immune response (1). Multiple populations of suppressive B cells have been described and defined by the expression of a variety of B-lineage and context-dependent markers (2), including transitional 2-marginal zone precursors (T2-MZP) (3), mature MZ B cells (4, 5), TIM-1 B cells (6), Plasmablasts (PBs) (7), Plasma Cells (PCs) (8), and B1a B cells (9). The immunosuppressive function of Bregs is primarily mediated by inhibition of T cell activation and pro-inflammatory responses (10). This may occur through direct or indirect mechanisms, including production of anti-inflammatory cytokines such as IL-10, IL-35, and transforming growth factor receptor β (TGF-β), inhibition through cognate interactions with pro-inflammatory immune cells, or by promoting Treg cell differentiation (11). Notably, the absence or loss of Bregs has been associated with increased inflammation in mice with a variety of autoimmune disorders, including experimental autoimmune encephalomyelitis (EAE) (12–14), ulcerative colitis (UC) (15, 16), collagen-induced arthritis (3), and SLE (17, 18). Despite evidence of an important role for suppressive B cells in modulating aberrant immune responses, however, their origins and regulatory mechanisms are poorly understood.

The important role of B cell regulatory function for maintaining immune tolerance has driven research on Bregs and their potential as targets for treatment of various autoimmune diseases, including SLE (19). However, deciphering the exact role of Bregs in SLE pathogenesis has been complicated by heterogeneity in lupus patients and Breg subsets described in the context of each study. Multiple studies have found increased serum IL-10 levels as well as increased numbers of circulating Bregs in SLE patients (20–22). However, others have described decreased Bregs in specific cohorts of SLE patients, including those with active lupus nephritis (LN) (23) and decreases in individual marker-defined Breg subsets (24, 25) indicating that specific Breg populations may be important for counteracting specific manifestations of SLE pathogenesis. Even when Bregs are present in sufficient numbers in SLE patients, they appear to be functionally impaired suggesting that defects in Breg-mediated suppression may contribute to autoimmunity (26–28). Thus, many questions remain as to the role of Bregs in SLE, including how specific Breg populations function in the context of autoimmunity and how this may be exploited to inform better treatment of SLE patients.

In previous work, we utilized the autoimmune-prone MRL/Mp-*Fas^lpr^* (MRL/*lpr*) mouse strain, which spontaneously develops lupus-like disease, to investigate the role of gut microbiota in autoimmunity (29, 30). Interestingly, early treatment with the antibiotic vancomycin before disease onset exacerbated disease pathogenesis and concomitantly lead to reduced numbers of splenic Bregs as well as decreased circulating IL-10 and IL-35. Moreover, adoptive transfer of Bregs pre-disease improved disease pathology, whereas transfer during active disease was not protective (30). This result emphasizes the importance of Bregs in preventing autoimmunity, but also suggests that these effects are disease-context dependent.

Here, we used a combination of phenotypic and single-cell transcriptional analysis of MRL/*lpr* mice as a model to examine the role of Bregs and heterogeneity of Breg subsets generated from mice at different stages of lupus disease progression. We identified deficiencies in the overall number of IL-10^+^ Bregs as well as differences in the proportion and transcriptional profiles of specific Breg subsets derived from mice with active disease as compared to mice before disease onset. This result suggests that not only deficiencies in Bregs as a whole, but alterations to the repertoire of Bregs may play a critical role in lupus pathogenesis and that enhancing the function of specific Breg subsets could be exploited to restore Breg-mediated suppression in the context of autoimmunity.

## Materials and methods

### Mice

MRL/*lpr* mice (MRL/Mp-*Fas^lpr^*, stock number 000485) were purchased from The Jackson Laboratory (Bar Harbor, ME) and kept in a pathogen-free facility per the requirements of Virginia Tech’s Institutional Animal Care and Use Committee (IACUC). Female MRL/*lpr* mice aged six weeks were used to represent the pre-disease stage and female MRL/*lpr* mice aged ten weeks were used to represent the active disease stage. Only female mice were used as these mice get earlier and more severe disease as compared to male mice in a manner similar to human patients with lupus and other autoimmune diseases.

### Flow cytometry

Spleens were collected and pressed through 70-μm cell strainers with complete medium (RPMI 1640, 10% fetal bovine serum, 1 mM sodium pyruvate, 1% 100 MEM non-essential amino acids, 10 mM HEPES, 55 mM 2-mercaptoethanol, 2 mM L-glutamine, 100 U/ml penicillin-streptomycin, all from Life Technologies, Grand Island, NY). Red blood cells (RBCs) were excluded using previously published methods (30). Single-cell suspensions were stimulated for 24 hours with 10 μg/ml LPS, followed by 5 hours of 50 ng/ml PMA and 500 ng/ml ionomycin, blocked for 10 minutes on ice with the Fc receptor block anti-CD16/32 (eBioscience), then stained for 15 minutes in the dark with fluorochrome-conjugated antibodies and analyzed with a BD FACS Aria II flow cytometer (BD Biosciences, San Jose, CA). Foxp3 Fixation/Permeabilization kit (eBioscience) was used for intracellular staining. To exclude dead cells, a Zombie Aqua fixable viability kit (Biolegend) was used. The following monoclonal anti-mouse antibodies were used in this study: AF700 or APC conjugated anti-CD19 diluted 1:800, PE-Cy7 conjugated anti-CD23 diluted 1:60, APC-Cy7 conjugated anti-CD21 diluted 1:80, FITC conjugated anti-CD24 diluted 1:200, PE conjugated anti-IL-10 diluted 1:80, BV711 conjugated anti-CD138 diluted 1:800, and APC conjugated anti-IgH (μ chain) diluted 1:800 (BioLegend).

### Cell preparation for analysis of Breg populations and IL-10 production

Enriched B cells were stimulated for 24 hours with 10 μg/ml LPS, followed by 5 hours of 50 ng/ml PMA and 500 ng/ml ionomycin. Stimulated cells were labeled with Miltenyi Biotec’s Regulatory B Cell Catch Reagent and incubated for 45 minutes. After removing the supernatant, the cells were resuspended in buffer and labeled with Regulatory B Cell Detection Antibody. Anti-PE microbeads were mixed into the cell suspension before magnetic separation on LS columns (Miltenyi Biotec) to capture IL-10 secreting Breg cells through positive selection.

### Cell preparation for single-cell RNA-seq

The splenocytes of three pre-disease female MRL/*lpr* mice were pooled into each of two samples (a total of 6 pre-disease mice) and the splenocytes of two active-disease female MRL/*lpr* mice were pooled into each of two samples (a total of 4 active-disease mice). Cells were stimulated for 24 hours with 10 μg/ml LPS, followed by 5 hours of 50 ng/ml PMA and 500 ng/ml ionomycin. IL-10 producing B cells were isolated using the Mouse Regulatory B Cell Isolation Kit purchased from Miltenyi Biotec (Gladbach, Germany) following the manufacturer’s protocol. In summary, single cell suspensions from spleens were enriched using the Regulatory B cell Biotin-Antibody cocktail, followed by the addition of Anti-Biotin MicroBeads and magnetic separation on LD Columns (Miltenyi Biotec).

### Single-cell RNA-seq

Single-cell RNA-seq was performed using 10X Genomics’ Chromium Single Cell 3′ V3.1 chemistry (Dual index). The experiment was designed to target 1,000 cells. Gel-Bead in Emulsions (GEMs) were made and the RT reaction was carried out in the Nexus GX2 PCR instrument (Eppendorf). Post-GEM RT-cleanup was used to obtain barcoded cDNA from the GEMs, which was then amplified for 12 cycles. According to the manufacturer’s protocol, amplified cDNA was subjected to enzymatic fragmentation, end-repair, A tailing, adaptor ligation, and 10X specific sample indexing. Bioanalyzer analysis was used to determine the quality and quantity of libraries. Following that, libraries were pooled and sequenced on the Illumina HiSeq platform (Novogene).

### Single-cell RNA-seq sample pre-processing, clustering, and dimensionality reduction

Sequencing reads were processed with 10x Genomics Cell Ranger software (v 7.0.1) using the standard pipeline. FASTQ files were generated and demultiplexed with the cellranger mkfastq command. Read alignment to mouse reference genome mm10 and gene quantification were carried out with cellranger count. Downstream analysis of output count, gene/feature, and barcode matrices was done using the R/BioConductor package Seurat (v 4.2.0). Quality control analysis was carried out to filter for high-quality cells with unique molecular identifier (UMI) counts per cell > 1000, genes per cell > 1000, log_10_ genes per UMI < 0.75, and ratio of cell reads from mitochondrial genes < 0.1 (all thresholds were set using empirical distributions). Doublet discrimination was carried out on filtered samples using DoubletFinder (v 2.0.3) and predicted doublets were removed for downstream analysis. Cell cycle scores were calculated using the CellCycleScoring function to ensure that cell cycle phase was not a significant source of variation in the data. Filtered datasets were split into Seurat objects corresponding to each cohort (pre-disease and active-disease) and count normalization and variance stabilization was carried out for each object using the SCTransform function. Integrated analysis was performed using the most highly variable shared genes to identify analogous populations and facilitate direct comparison of differential gene expression (DEG) between cohorts. Cell clustering was performed with Seurat using the top 15 principal components (PCs) as determined by the elbow method with a cluster resolution of 0.8 for separate cohort analysis and resolution of 0.4 for integrated analysis. Clusters were visualized with the Uniform Manifold Approximation and Projection (UMAP) method. Cluster markers were identified using the FindAllMarkers function with logfc.threshold of 0.5 for separate cohort clusters and of 0.25 for integrated clusters. DEGs in active-disease cells from integrated clusters were identified using the FindMarkers function with the Wilcoxon rank sum test. DEGs with p<0.05 were considered statistically significant.

### Single-cell cluster annotation

Single-cell clusters were annotated based on enrichment of cluster marker genes with pre-defined Breg subset and immune/cellular pathway gene sets listed in **Supplementary Table 1.** Curated gene sets were generated based on a combination of literature mining, Mouse Genome Informatics (MGI) gene ontology (GO) terms, and immune cell-specific expression compiled from the Immunological Genome Project Consortium (ImmGen). Enrichment statistics for overlap of cluster markers with curated gene sets were calculated by a two-sided Fisher’s Exact Test in R using the function fisher.test(). Enrichments with p<0.05 were considered statistically significant. The unannotated clusters, representing populations with cluster-defining marker genes that were not informative in identifying specific Breg subsets, were removed from downstream analyses.

### Ingenuity pathway analysis

The canonical pathway function of IPA core analysis (Qiagen) was used as an independent method of annotated differentially expressed genes (DEGs) between active-disease and pre-disease IL-10^+^ B cells within integrated single-cell clusters. Overlap p-values of p<0.05 were considered statistically significant.

### Single-cell pseudotime trajectory analysis

Trajectory analysis was carried out to estimate developmental relationships between the Seurat-generated IL-10^+^ B cell clusters from pre-disease and active-disease MRL*/lpr* mice using the R/Bioconductor package Monocle 3 (v 1.3.1). Trajectories were constructed using the learn_graph() function and cells were ordered in pseudotime using the IgD^mid^ Naïve B Cell cluster (cluster 6) as the root cluster for pre-disease cells and the Naïve B Cell Cluster (cluster 2) as the root cluster for active-disease cells. Cells were visualized based on pseudotime in a trajectory heatmap using the plot_cells() function and displayed as boxplots depicting the range of pseudotime values for each cluster using ggplot().

### Gene set variation analysis (GSVA)

Gene sets used as input for GSVA are listed in **Supplementary Table 1**. The R/Bioconductor package GSVA (31) (v1.36.3) was used as a non-parametric method to estimate variation in enrichment of these gene sets in publicly available microarray data from isolated murine CD138^+^ PB-PCs (GSE103458) as previously described (32).

### Statistical analysis

Enrichment statistics (p-values and odds ratios) for the overlap of single-cell cluster markers and pre-defined gene sets were calculated using a two-sided Fisher’s Exact Test in R with confidence level of 0.95. Statistical tests and graphs comparing GSVA enrichment scores for isolated CD138^+^ IL-10^+^ and IL-10^-^ cells were calculated using an unpaired, two-sided Welch’s t-test in GraphPad Prism (v9.3.1).

### Study approval

The study was approved by the Virginia Tech IACUC under protocol number 21-003.

## Results

### Bregs are numerically and functionally impaired in lupus-prone mice with active disease

**Figure 1:**
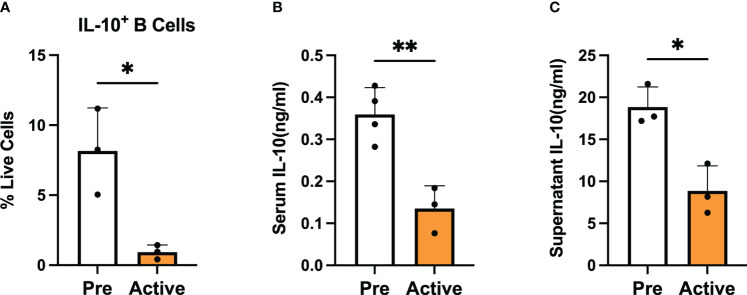
Bregs are numerically and functionally impaired in lupus-prone mice with active disease. Female MRL*/lpr* mice were assessed at the pre-disease (6-8 weeks) and active-disease (10-12 weeks) stages. Splenic IL-10 producing B cells were generated by stimulation with 10 μg/ml LPS for 24 h followed by 5 h of 50 ng/ml PMA and 500 ng/ml ionomycin. **(A)** Percentage of splenic IL-10 producing B cells. **(B)** Serum level of IL-10. **(C)** Supernatant level of IL-10 from stimulated splenic B cells. *p < 0.05, ** *p* < 0.01.

To investigate the role of regulatory B cells (Bregs) in the establishment and progression of disease in MRL/*lpr* mice, we chose to focus on differences in the number and suppressive function of Bregs generated from mice at two disease stages: before disease onset (pre-disease; 6-8 weeks) and after establishment of autoimmunity (active-disease; 10-12 weeks). Therefore, splenic B cells were isolated from pre-disease and active-disease mice, stimulated with 10 μg/ml LPS for 24 h followed by 5 h of 50 ng/ml PMA and 500 ng/ml ionomycin and the numbers of IL-10^+^ B cells and IL-10 production were assessed. The frequency of splenic IL-10^+^ B cells was significantly decreased in mice with active disease as compared to the pre-disease stage (**Figure 1A**). In addition, levels of serum IL-10 were significantly decreased in active-disease mice (**Figure 1B**). To identify potential deficiencies in IL-10 mediated Breg suppression during the active-disease stage, levels of IL-10 were assessed in the supernatant of *in vitro* stimulated splenic B cells. As with total serum IL-10 levels, production of IL-10 by B cells from mice with active disease was also significantly reduced (**Figure 1C**). Overall, these results indicate that the development of autoimmunity in lupus-prone mice is accompanied by both numerical and functional defects in regulatory B cells.

### scRNA-seq analysis identifies Breg cell subsets in lupus-prone mice at the pre-disease stage

To define variations in Bregs from lupus-prone mice at different stages of disease progression, we carried out single-cell RNA sequencing (scRNA-seq) on IL-10^+^ CD19^+^ B cells generated from spleens of female MRL/*lpr* mice at the pre-disease stage (6-8 weeks old) or active-disease stage (10-12 weeks old). To account for the increased expansion of splenic B cells in active-disease mice, IL-10^+^ B cells were pooled from 3 pre-disease mice or 2 active-disease mice per sample to yield approximately 10,000 total cells in duplicate for each disease cohort. Quality control and doublet discrimination were carried out separately on each sample before singlets from each disease stage were combined to yield a final total of 12,130 pre-disease and 13,895 active-disease cells retained for downstream analyses.

**Figure 2:**
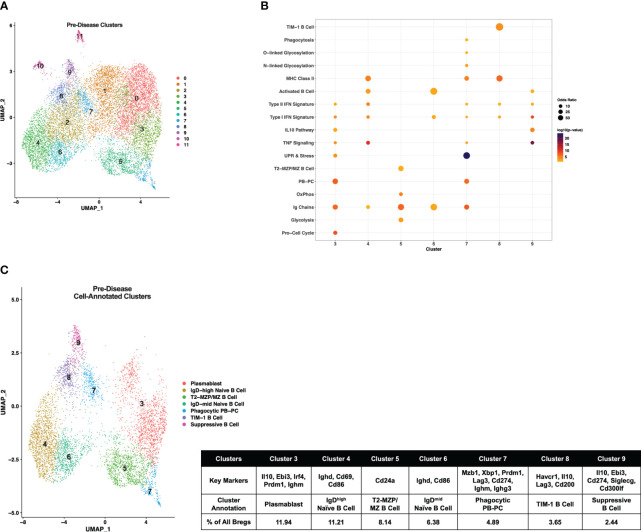
Single-cell transcriptional analysis identifies subsets of IL-10^+^ Bregs from lupus-prone mice prior to disease onset. Splenic IL-10 producing B cells (Bregs) were generated from pre-disease female MRL*/lpr* mice and analyzed by single-cell RNA-seq. **(A)** UMAP plot showing 12 clusters of pre-disease Bregs. **(B)** Bubbleplot depicting the overlap of pre-disease Breg cluster markers with pre-defined cell type and pathway gene sets. **(C)** Annotation of 7 pre-disease clusters that had significant overlap of cluster marker genes with Breg subset gene sets. Key markers used to define each cluster annotation and percentage of cells in each cluster out of total Bregs are displayed.

Initially, pre-disease and active-disease cells were evaluated separately to identify groups of Breg cells with shared transcriptional profiles from lupus-prone mice at each stage of disease progression. IL-10^+^ B cells from pre-disease mice formed 12 cell clusters (**Figure 2A**), that were annotated based on enrichment of cluster-defining gene markers in pre-defined gene sets representative of previously identified Breg subsets, cellular processes, and inflammatory pathways **Supplementary Table 1**. As a result, we identified that 7 of the 12 clusters had significant cluster marker overlaps with Breg subset genes indicating that these clusters represented transcriptionally distinct subpopulations of IL-10^+^ B cells from pre-disease lupus mice (**Figure 2B**). These clusters were selected for further investigation and unannotated clusters, in which cluster markers were not informative in defining Breg subsets, were removed. The combined cell type and pathway gene set enrichments and key cluster marker genes were then used to label each annotated Breg subpopulation (**Figure 2C**; **Supplementary Figure 1**). The highest proportion of annotated Breg cells (11.94%) in cluster 3 were designated as Plasmablasts (PBs) by enrichment of the Plasmablast/Plasma Cell (PB-PC), Ig Chain, Pro-Cell Cycle, and UPR & Stress gene sets, including increased expression of *Irf4, Prdm1, Ighm, Il10*, and *Ebi3*. Two populations in clusters 4 and 6 of pre-disease cells were representative of naïve B cells, although they also manifested enrichment of the Activated B Cell gene set and increased expression of *Ighd* relative to other clusters. As cluster 4 had the highest *Ighd* expression, these cells were designated IgD^high^ Naïve B Cells and cluster 6 cells as IgD^mid^ Naïve B Cells. Pre-disease Bregs in cluster 5 were enriched for markers of T2-MZP/MZ B Cells, including *Cd24a* as well as metabolic pathway genes involved in Glycolysis and Mitochondrial Oxidative Phosphorylation (OxPhos). Cluster 7 cells were enriched for gene sets for PB-PCs (*Mzb1, Xbp1, Prdm1*), Ig Chains (*Ighm, Ighg3*), MHC Class II, N- and O-linked Glycosylation, and UPR & Stress indicative of a mixed population of PBs and class-switched PCs. Interestingly, this cluster was also enriched for the Phagocytosis gene set and thus was designated as the Phagocytic PB-PC subset. Because of the high expression of *Havcr1* as compared to other clusters, cluster 8 cells were labeled TIM-1 B Cells. Finally, the lowest proportion of annotated Bregs in pre-disease mice was a population of activated B cells that expressed the highest levels of *Il10* as compared to other clusters in conjunction with other anti-inflammatory molecules, including *Ebi3, Cd274* encoding PD-L1, *Siglecg*, and *Cd300lf* and, therefore, was designated Suppressive B Cells.

### Transcriptionally-defined IL-10^+^ B cell subsets are altered in lupus-prone mice with active disease

**Figure 3:**
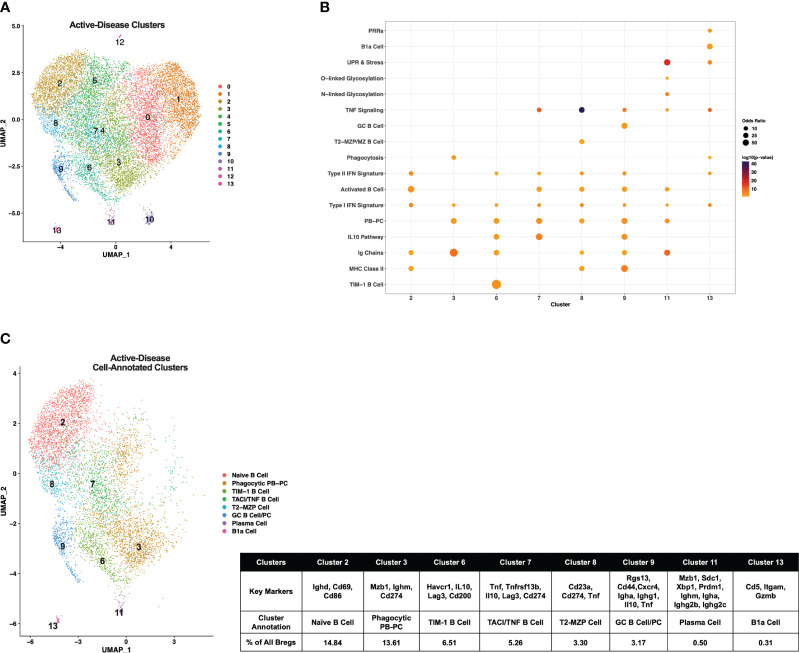
Single-cell analysis reveals differences in transcriptional profiles IL-10^+^ Bregs present in mice with ongoing autoimmune inflammation. Splenic Bregs were generated from active-disease female MRL*/lpr* mice and analyzed by single-cell RNA-seq. **(A)** UMAP plot showing 14 clusters of active-disease Bregs. **(B)** Bubbleplot depicting the overlap of active-disease Breg cluster markers with pre-defined cell type and pathway gene sets. **(C)** Annotation of 8 active-disease clusters that had significant overlap of cluster marker genes with Breg subset gene sets. Key markers used to define each cluster annotation and percentage of cells in each cluster out of total Bregs are displayed.

Single-cell gene expression profiles of IL-10^+^ B cells from active-disease mice exhibited a distinct spatial pattern from pre-disease mice that resulted in 14 clusters of cells in similar molecular states (**Figure 3A**), 8 of which were enriched for pre-defined B cell subset markers (**Figure 3B**). Overall, cluster enrichments in active disease reflected a heightened inflammatory state with an increased number of clusters enriched for Type I/II IFN signature genes with or without enrichment of TNF signature genes as compared to pre-disease mice (**Figure 3B**). Cluster annotations for Breg cells from active-disease mice also exhibited key variations from pre-disease mice indicative of the more pro-inflammatory environment present during active autoimmunity (**Figure 3C**; **Supplementary** **Figure 2**). Several of the Breg clusters found in pre-disease mice were also present during active disease, but in differing proportions of the total analyzed population, and we found key variations in markers defining these subsets. Unlike in pre-disease mice, the Naïve B Cell cluster of active-disease cells expressing *Ighd* (cluster 2) was confined to one cluster and represented the greatest proportion of total Bregs (14.84%). The Breg cluster enriched for the PB-PC and Phagocytosis gene signatures (cluster 3) was present at a greater percentage (13.61%) in active-disease as compared to pre-disease (4.89%) mice and unlike in pre-disease mice, appeared to be a pre-class switched population expressing *Ighm* and was also the lone active-disease cluster with *Cd274* as a significant cluster marker. The TIM-1 B Cell cluster (cluster 6) was more highly represented in active-disease mice (6.51%) whereas the T2-MZP Cell cluster (cluster 8) was less represented (3.30%) and appeared more skewed to a MZP population through increased expression of *Cd23a* as compared to other clusters.

Two clusters unique to IL-10^+^ cells from active-disease mice were annotated with GC B Cell (cluster 9) and/or PC markers (cluster 11). Cluster 9 cells were identified as GC B Cells/PCs because of expression of *Rgs13* as well as *Igha* and *Ighg1* and cluster 11 cells were labeled PCs because of expression of *Sdc1, Ighm, Igha*, and *Ighg2b/c*. This was particularly notable as in pre-disease mice the only cells with evidence of class-switching were found in the Phagocytic PB-PC cluster and expressed *Ighg3*. The final two cell marker-annotated clusters of active-disease Bregs (cluster 7 and cluster 11) were characterized by expression of unique inflammatory signatures and markers. Cluster 7 was enriched for the IL-10 Pathway and TNF Signaling gene signatures and had increased expression of *Tnfrsf13b* encoding the B cell survival factor receptor TACI and thus was designated as the TNF/TACI B cell cluster. Finally, cluster 11 represented a minor outlier population (0.31%) of innate-like B1a cells expressing *Cd5, Itgam*, and *Gzmb* and enriched for the Pattern Recognition Receptor (PRR) and UPR & Stress gene signatures. Thus, gene expression-derived Breg subsets from lupus-prone mice with active disease were more represented by PB-PC populations than in pre-disease mice and had a lower proportion of MZ-like cells expressing markers indicative of a more naïve developmental stage.

### Single-cell trajectory analysis suggests developmental relationships among Breg subsets from lupus-prone mice at pre-disease and active-disease stages

**Figure 4:**
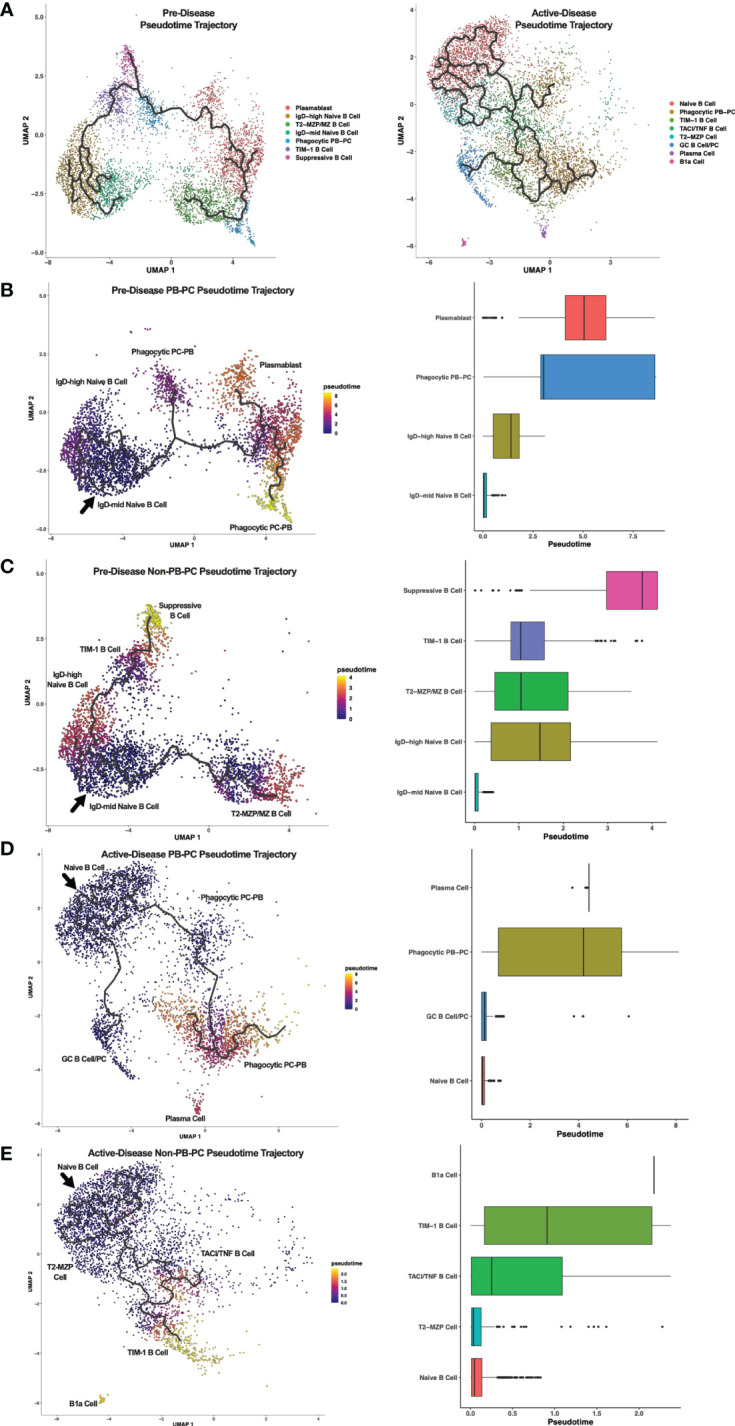
Trajectory analysis reveals relationships between Breg subsets from lupus-prone mice at pre-disease and active-disease stages. Breg subset marker-annotated single-cell clusters from pre-disease and active-disease mice were ordered in pseudotime using Monocle 3. **(A)** Trajectory plots of pre-disease and active-disease Breg clusters. **(B, C)** Trajectory mapping of pre-disease plasmablast/plasma cell (PB-PC) and non-PB-PC annotated clusters. Clusters were ordered in pseudotime distance originating from the IgD-mid Naïve B Cell annotated cluster. **(D, E)** Trajectory mapping of active-disease PB-PC and non-PB-PC annotated clusters. Clusters were ordered in pseudotime distance originating from the Naïve B Cell annotated cluster. Boxplots show the pseudotime range of each Breg cluster in relation to the root cluster.

Breg cells are generally defined by expression of IL-10, but it is unclear how Bregs are induced and whether or how Breg subsets are developmentally linked (1). In addition, it is not known how Breg development or differentiation may be altered in the context of autoimmunity. To investigate the relationships among Breg subsets and to compare differences in populations identified at different stages of disease progression in lupus-prone mice, we carried out single-cell trajectory analysis to order IL-10^+^ B cells in pseudotime based on changes in gene expression (**Figure 4A**). Initially, we noted the increase in overall complexity of cellular trajectories between active-disease as compared to pre-disease cells which could arise from the greater variety of inflammatory stimuli inducing B cell production of IL-10. Because of the critical role of autoantibody producing PCs in lupus disease pathology, Breg subsets from pre-disease and active-disease mice were divided into PB-PC and non-PB-PC annotated clusters. Learned trajectories for each group of clusters were created and rooted in the Naïve B cell cluster as we predicted this subset to be the least developmentally advanced B cell population at each disease stage. Then other clusters were ordered by relative pseudotime distance along the trajectory from the root population and displayed as heatmaps detailing the trajectory paths and boxplots depicting the range of pseudotime encompassed by each Breg cluster. From pre-disease mice, the PB-PC annotated clusters followed three branching trajectories stemming from the Naïve B cell cluster (**Figure 4B**). The Phagocytic PB-PC population exhibited a wide pseudotime range that was split into two groups, one of less differentiated cells likely representing PBs and one of highly differentiated cells likely representing class-switched PCs. In contrast, the PB cluster from pre-disease appeared to be in an intermediate developmental state between the two subsets of Phagocytic PB-PC cells. The Non-PB-PC pre-disease clusters followed two distinct developmental trajectories stemming from the IgD^mid^ Naïve B Cell cluster (**Figure 4C**). Along one trajectory were the T2-MZP/MZ B cells, which represented a range of pseudotimes representing the differentiation from a precursor population to a mature IL-10^+^ MZ B cell. Along the second trajectory, the TIM-1 B cell cluster appeared to be more proximal in pseudotime to naïve cells as compared to the Suppressive B cell population which was the furthest cluster along the pseudotime trajectory in pre-disease mice.

Single cell trajectory analysis of IL-10^+^ B cells from mice with active disease revealed notable differences in Breg developmental states as compared to pre-disease mice. Notably, trajectories in active-disease mice were much more complex. Anchored in the Naïve B Cell cluster, PB-PC annotated clusters formed two branching trajectories (**Figure 4D**). Similar to pre-disease mice, the Phagocytic PB-PC population of active-disease cells was divided into a less differentiated, early subpopulation representing PBs and a more differentiated, late subpopulation representing PCs. However, unlike pre-disease mice, we also observed a second trajectory of GC B cells and class-switched PCs indicative of a robust and ongoing production of autoantibody producing cells during the height of disease activity. Trajectory analysis of Non-PB-PC, active-disease IL-10^+^ B cells was carried out to predict the developmental pathways of other unique Breg subets present in mice with ongoing disease pathology (**Figure 4E**). Interestingly, as suggested by the differences in cluster marker expression, the T2-MZP cell cluster in active-disease mice appeared to be a less differentiated population nearly identical to the Naïve B cell cluster in pseudotime and with no indication of a more developed population of MZ B cells as observed in pre-disease mice. More advanced populations in pseudotime exhibited a split trajectory between the TACI/TNF B cell cluster and the TIM-1 B cell cluster that included cells with a wide pseudotime range indicative of within-cluster heterogeneity in gene expression profiles. Finally, the outlier cluster of B1a cells, unique to active-disease mice, was the most advanced in pseudotime as compared to the naïve population and appeared to be developmentally distinct and unlinked to the developmental trajectory of other Breg subsets. Thus, pseudotime trajectory analysis of IL-10^+^ B cells from lupus-prone mice revealed multiple pathways leading to the development of transcriptionally diverse Breg subsets present at different stages of disease progression.

### Integrated single-cell analysis of pre-disease and active-disease lupus-prone mice identifies stage-dependent alterations in IL-10^+^ B cell populations

**Figure 5:**
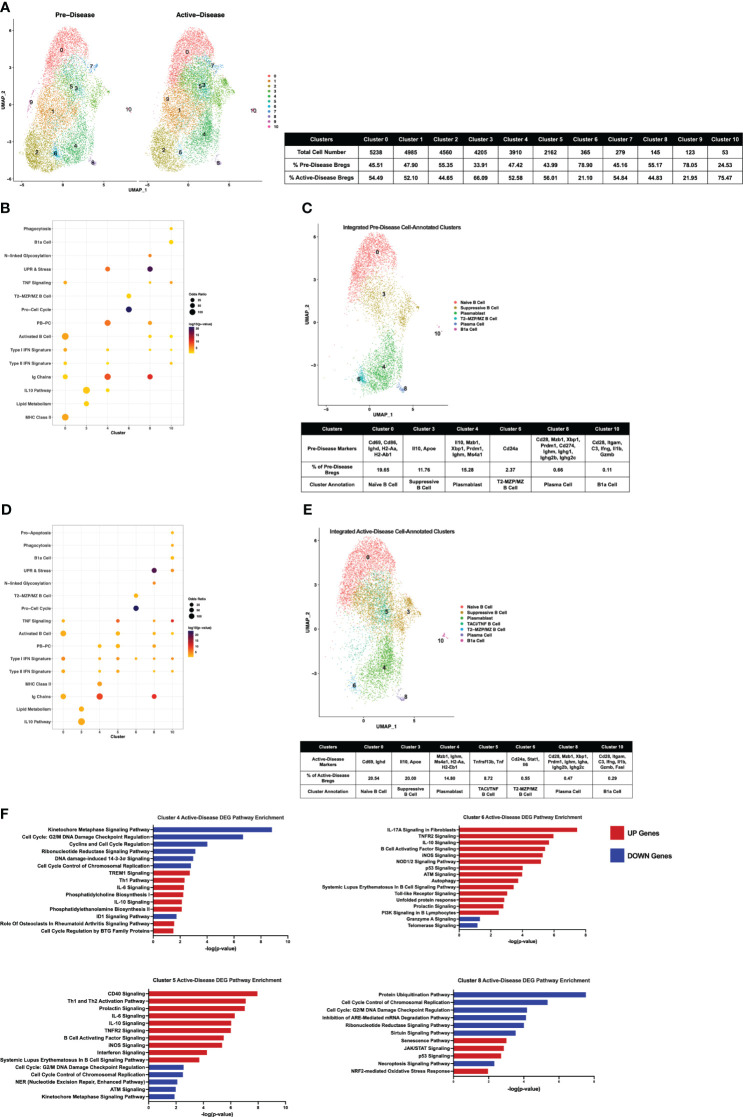
Single-cell integration compares analogous Breg subsets present through stages of disease progression in lupus-prone mice. Single-cell datasets of Bregs from pre-disease and active-disease mice were integrated and co-clustered to facilitate comparison of cells with shared biological states. **(A)** Split UMAP plots of 11 integrated clusters of pre-disease and active-disease Bregs. **(B, D)** Bubbleplots depicting the overlap of integrated pre-disease **(B)** and active-disease **(D)** Breg clusters with pre-defined cell type and pathway gene sets. **(C, E)** Annotation of 6 pre-disease **(C)** and 7 active-disease **(E)** integrated clusters exhibiting significant overlap of cluster marker genes with Breg subset gene sets. Key markers used to define each cluster annotation and percentage of cells in each cluster out of total Bregs are displayed. **(F)** IPA canonical pathway analysis of differentially expressed genes in active-disease Bregs as compared to pre-disease Bregs from selected integrated single-cell clusters.

To compare IL-10^+^ B cells generated from lupus-prone MRL/*lpr* mice at different stages of disease progression directly, scRNA-seq datasets of pre-disease and active-disease cells were integrated and co-clustered based on shared sources of transcriptional variation (**Figure 5A**). With this approach, each integrated cluster represents a population of Breg cells in a shared biological state between pre-disease and active-disease mice. Then, cluster markers were identified separately from pre-disease and active-disease cells within each integrated cluster in order to highlight differences in co-clustered Breg cells generated from each disease stage, and used to assign Breg subset annotations. Integrated cluster markers derived from pre-disease cells identified 6 clusters with significant marker overlaps with pre-defined Breg subset genes and cellular pathways that were used to assign each cluster identity (**Figures 5B, C**. The highest proportion of pre-disease cells (19.65%) in cluster 0 represented Naïve B cells with expression of *Ighd*, the Activated B Cell markers *Cd69* and *Cd86*, and MHC Class II genes *H2-Aa* and *H2-Ab1* (**Figure 5C**; **Supplementary** **Figure 3**). Cluster 3 contained 11.76% of pre-disease Bregs and was designated the Suppressive B Cell cluster as these cells expressed the highest levels of *Il10* and, interestingly, were also enriched for the Lipid Metabolism gene set, including high expression of *Apoe*. The PB annotated cluster (cluster 4) consisted of 15.78% of pre-disease cells and was designate PB based on enrichment of the PB-PC (*Mzb1, Xbp1, Prdm1, Ms4a1*) and Ig Chains (*Ighm*) gene sets. Pre-disease Bregs in cluster 6 (2.37%) represented T2-MZP/MZ B cells with high expression of *Cd24a* and were also highly enriched for Pro-Cell Cycle genes. The final two cell-annotated integrated clusters represented minor populations of pre-disease Bregs including 0.66% of cells in cluster 8 annotated as PCs and the outlier population of B1a cells (0.11%) in cluster 10. Pre-disease PCs were enriched for Activated B cell (*Cd28*), PB-PC (*Mzb1, Xbp1, Prdm1, Cd274*), Ig Chains (*Ighm, Ighg1, Ighg2b, Ighg2c*), N-linked glycosylation, and UPR & Stress gene sets, whereas pre-disease B1a cells were marked by high expression of the pro-inflammatory markers *Itgam, C3, Ifng, Il1b*, and *Gzmb*.

Integrated cluster marker enrichment analysis and annotation of active-disease IL-10^+^ B cells resulted in shared labels for the 6 clusters of pre-disease cells, but with key differences in the proportion of total cells and marker genes in each cluster (**Figures 5D, E**). The Naïve B cell cluster (cluster 0) consisted of a similar proportion of total active-disease cells (20.54%) as compared to pre-disease cells and was still characterized by enrichment of the Activated B cell markers *Cd69* and *Ighd*, but unlike pre-disease Naïve B cells, active-disease B cells were not enriched for MHC Class II genes. Active-disease Suppressive B cells in cluster 3 shared representative cluster markers with pre-disease cells, but contained a higher proportion of total cells (20% *vs*. 11.76%). In contrast, a lower proportion of active-disease cells were PBs in cluster 4 and further differed from analogous pre-disease cells through enrichment of MHC Class II genes (*H2-Aa, H2-Eb1*), markers of the Naïve B cell cluster of pre-disease cells. A lower proportion of active-disease cells as compared to pre-disease cells were found in the T2-MZP/MZ B cell annotated cluster (cluster 6, 0.55% *vs*. 2.37%) and active-disease cells in this cluster also uniquely expressed marker genes *Stat1* and *Il6.* The PC cluster of active-disease Bregs in cluster 8 was a modestly smaller proportion of total cells than in pre-disease (0.47% *vs*. 0.66%) and exhibited differences in expression of class-switched Ig Chain genes, including *Igha* in place of *Ighg1* on pre-disease cells and were not enriched for expression of *Cd274.* In contrast, the B1a cell-annotated cluster 10 represented a larger proportion of total active-disease cells (0.29% *vs*. 0.11%) and further differed in expression of *Fasl* as a cluster marker.

We also noted that a new, cell-annotated, integrated cluster emerged from active-disease IL-10^+^ B cells that was not observed in pre-disease mice. Active-disease Bregs in cluster 5 exhibited increased expression of Activated B cell markers as compared to other clusters and were further distinguished by enrichment of the PB-PC and TNF Signaling gene sets (**Figure 5E**; **Supplementary** **Figure 4**). Key marker genes defining this cluster included increased expression of *Tnf* and *Tnfrsf13b* encoding TACI and thus this cluster was designated the TACI/TNF B cell cluster. Overall, integrated clustering and separate cluster marker identification of pre-disease and active-disease IL-10^+^ B cells in similar biological states revealed key variations in analogous populations of regulatory B cells through disease progression in lupus-prone mice.

### Transcriptomic comparison of integrated single cell datasets reveals pathologic inflammatory gene signatures associated with IL-10^+^ B cells from active-disease mice

To evaluate differences in gene expression profiles between co-clustered populations of pre-disease and active-disease IL-10^+^ B cells, we identified differentially expressed genes (DEGs) in active-disease Bregs as compared to their pre-disease counterparts within the same integrated cluster. For this analysis, we focused on 4 of the 6 clusters found in both active-disease and pre-disease mice. These included cluster 4 PBs, cluster 5 TACI/TNF B cells, cluster 6 T2-MZP/MZ B cells, and cluster 8 PCs. For each cluster, up and down-regulated genes in active-disease cells were used as input for Ingenuity Pathway Analysis (IPA) to identify significant overlaps with canonical pathways (**Figure 5F**). As a whole, we found that active-disease Bregs exhibited increased expression of genes involved in inflammatory and stress response pathways and decreased expression of genes involved in cell cycle and DNA repair pathways. Specifically, in cluster 4 PBs from mice with active disease, up-regulated DEGs were enriched for TREM1 Signaling, Th1 response, IL-6 signaling, and phospholipid synthesis, whereas down-regulated DEGs were enriched for cell cycle regulation and DNA damage response pathways (**Figure 5F**). Active-disease cells in the TACI/TNF cluster (cluster 5) manifested increased expression of several immune activation pathways and pro-inflammatory signaling pathways through CD40, IL-6, TNFR2, B Cell Activating Factor, iNOS, and IFN. Like the PB cluster, active-disease cells in the TACI/TNF cluster had decreased expression of genes involved in DNA damage repair and cell cycle arrest pathways. In addition to inflammatory mediator signaling pathways, DEGs in active-disease cells in the T2-MZP/MZ B cell cluster (cluster 6) were indicative of cellular dysfunction and stress with involvement in pathways including autophagy, the unfolded protein response, p53 signaling, and telomerase signaling. Finally, DEGs from cluster 8 PCs were largely decreased in active-disease mice and were enriched for pathways related to cell cycle checkpoint control and metabolic regulation, including protein ubiquitination, mRNA degradation, and sirtuin signaling. Conversely, upregulated genes in active-disease cluster 8 PCs were involved in cellular senescence and the oxidative stress response (**Figure 5F**). Notably, we found that expression of *Il10* and IL-10 Signaling Pathway genes were consistently upregulated in active-disease Bregs from all clusters (**Supplementary** **Figure 5**), indicating that pathologic autoimmunity in these mice was not related to a deficiency in expression of *Il10* transcript, but rather a separate source of regulatory dysfunction. Thus, DEGs from IL-10^+^ B cells in the context of active autoimmunity were indicative of response to a heightened inflammatory environment resulting in immune activation and exposure to intra- and extra-cellular stressors that could affect the ability of these cells to function as immune regulators.

### Validation of transcriptionally defined alterations to the landscape of Breg subsets in lupus-prone mice with active disease

**Figure 6:**
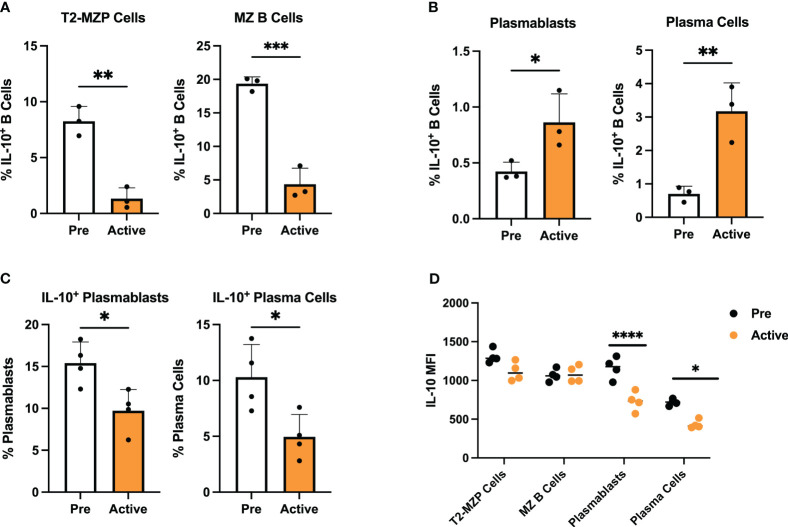
Transcriptionally defined disease-dependent alterations to Breg subsets are confirmed in lupus-prone mice. Splenic Breg subsets generated from female MRL*/lpr* mice were assessed at the pre-disease (6-8 weeks) and active-disease (10-12 weeks) stages. **(A)** Percentage of T2-MZP cells and MZ B cells from total IL-10^+^ cells. **(B)** Percentage of PBs and PCs from total IL-10^+^ cells. **(C)** Percentage of IL-10 producing PBs and IL-10 producing PCs from total PBs and total PCs. **(D)** Mean fluorescence intensity (MFI) of IL-10 expression from splenic Breg subsets in A-C. *p < 0.05, **p < 0.01, ***p < 0.001, ****p < 0.0001.

To confirm the changes we observed in representation of specific Breg subsets using single-cell gene expression analysis, we carried out flow cytometric staining of splenic IL-10^+^ B cells from MRL/*lpr* mice at the pre-disease or active-disease stages. We focused on changes in the proportion of MZ and PB-PC lineage cells, as these populations showed the greatest difference based on expression of canonical marker genes. Notably, both T2-MZP (CD19^+^IL10^+^CD21^+^CD24^hi^IgM^+^CD23^+^) and MZ B cells (CD19^+^IL10^+^CD21^+^CD24^hi^IgM^+^CD23^-^) were significantly decreased in the spleens of active-disease as compared to pre-disease mice (**Figure 6A**; **Supplementary** **Figure 6**). In contrast, we observed significant increases in the proportion of splenic PBs (CD19^+^CD138^+^) and PCs (CD19^-^CD138^+^) in active-disease mice (**Figure 6B**; **Supplementary** **Figure 6**). Both of these results were in line with differences in the proportion of cells expressing a T2/MZP/MZ B cell or PB-PC gene signature as determined by scRNA-seq. However, despite increases in total PB-PCs in active-disease mice, the percentage of both PBs and PCs expressing IL-10 was significantly decreased (**Figure 6C**; **Supplementary** **Figure 6**). This result suggests that the the imbalance between pathogenic and regulatory, IL-10 producing PB-PCs contributes to the progression of autoimmune disease in lupus-prone mice.

Our initial measurement of serum IL-10 and IL-10 produced by activated IL-10^+^ B cells indicated that both were significantly decreased in lupus-prone mice with active disease. However, expression of *Il10* and IL-10 signaling pathway transcripts were consistently upregulated in active-disease Bregs from integrated single cell clusters. To investigate this further, we carried out intracellular staining for IL-10 in isolated MZ and PB-PC lineage B cells from pre-disease and active-disease mice (**Figure 6D**). As a result, we found no difference in mean fluorescent intensity (MFI) of IL-10 in T2-MZP or MZ B cells based on disease stage. However, in line with the decreased secreted IL-10 from total splenic Bregs, both PBs and PCs from active-disease mice expressed significantly reduced levels of intracellular IL-10. Therefore, this result demonstrates that the dominant populations of IL-10 producing B cells present in active-disease mice also display the greatest evidence of deficiency in IL-10 mediated immunoregulation.

**Figure 7:**
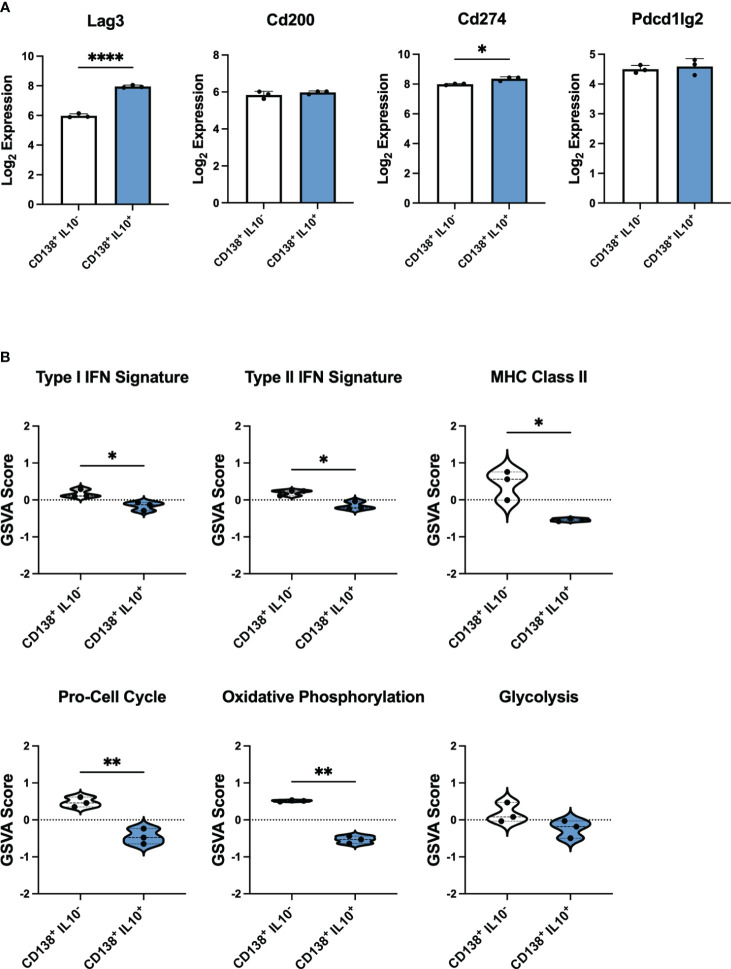
IL-10 producing PB-PCs induced by infection differ in inflammatory gene expression from those derived from autoimmune mice. Gene expression analysis by bulk RNA-seq of splenic CD138^+^IL10^-^ and CD138^+^IL10^+^ cells isolated from mice 24 hours after bacterial infection. **(A)** Log_2_ expression values of selected inhibitory receptor genes. **(B)** Gene set variation analysis (GSVA) comparing enrichment of inflammatory and cellular pathway gene sets between IL10^-^ and IL10^+^ PB-PCs from infected mice. *p < 0.05, ** *p* < 0.01, **** *p* < 0.0001.

To explore the differences in IL-10 producing PB-PCs from mice with active lupus that could render these cells unable to suppress autoimmunity effectively, we sought to establish a baseline of gene signatures expressed by Bregs from the PB-PC lineage in a normal immune response. To achieve this, we analyzed publicly available bulk gene expression data from a population of IL-10 producing PB-PCs induced in response to bacterial infection (33). These cells were initially characterized as CD138^+^IL10^+^ cells that exhibited increased expression of the inhibitory receptors LAG3, CD200, PD-L1, and PD-L2 as compared to CD138^+^IL10^-^ cells and were the predominant source of IL-10 produced in response to infection. Our analysis of these mice confirmed that log_2_ gene expression of *Lag3* and *Cd274* were significantly increased in CD138^+^IL10^+^ cells (**Figure 7A**). Interestingly, we found similar populations of IL-10 producing cells from scRNA-seq of lupus-prone mice as these markers were expressed by the Phagocytic PB-PC and TIM-1 B Cell populations from pre-disease mice and the TACI/TNF B Cell and TIM-1 B Cell populations from active-disease mice. Next, to characterize the immune profiles of CD138^+^IL10^+^ PB-PCs induced in a normal response to infection, we carried out gene set variation analysis (GSVA) with pre-defined gene sets comprising cellular and inflammatory pathways (**Figure 7B**). As a result, we found that CD138^+^IL10^+^ cells were de-enriched for inflammatory signatures of Type I IFN, Type II IFN, and MHC Class II and also exhibited decreased enrichment of proliferation and metabolism-related signatures as compared to CD138^+^IL10^-^ cells. Notably, this was in contrast to gene expression profiles of IL10-producing PB-PCs from lupus-prone mice, which were enriched for pro-inflammatory gene signatures and thus appeared more like IL10^-^ cells from mice post-infection. This result suggested that differences in Breg induction in the context of autoimmunity as compared to a normal immune response may alter the anti-inflammatory nature of IL-10 producing cells. Overall, we have demonstrated that disease stage-dependent differences in the single-cell transcriptional profiles of IL-10^+^ B cells directly translate to differences in the proportion of these subsets present in lupus-prone mice and that this likely contributes to their inability to control the onset or severity of autoimmunity.

## Discussion

Deficiencies in number and/or function of IL-10 producing, suppressive B cells or Bregs have been found both in mouse models of SLE and human SLE patients suggesting an important role for Bregs in controlling autoimmunity (34). However, because of the heterogeneity in subset markers and mechanisms of suppression, it remains unclear how Bregs are phenotypically and functionally altered in lupus and how this contributes to disease pathology. In this study, we utilized single-cell RNA sequencing to identify disease stage-dependent changes to the transcriptional profiles of Bregs generated from lupus-prone mice that were further validated by phenotypic analyses. As a result we have identified specific Breg subsets and inflammatory gene signatures associated with active disease that provide insights into the role of Bregs in lupus pathogenesis.

Our past work revealed that Bregs isolated from lupus-prone MRL/*lpr* mice before disease onset, but not Bregs from mice with active disease, were able to attenuate autoimmunity suggesting that active-disease Bregs were functionally impaired (30). In the present work, we confirmed that numbers of Bregs, levels of total serum IL-10, and production of IL-10 by Bregs induced from mice with active disease were all significantly decreased as compared to pre-disease mice. This result is in line with numerous studies that have demonstrated associations between numerical and functional impairment of Bregs and increased risk for development or enhanced severity of murine models of autoimmune disease (13, 14, 17, 28). However, these studies did not investigate the relative roles of different subsets of Bregs and their contributions to controlling or exacerbating disease.

We previously demonstrated the utility of gene expression analysis by bulk RNA-seq to construct immune profiles of lupus-prone mice and directly translated these results to human lupus patients (32, 35). Single-cell sequencing technology offers an advantage over bulk sequencing when studying rare cell populations or identifying and comparing subsets of select cell populations, such as Bregs (36). A previous study carried out scRNA-seq to characterize Bregs in different murine organs, but did not isolate IL-10 producing cells before sequencing and instead relied on marker genes to separate Bregs from total B cells (37). Previous studies have also used scRNA-seq to profile the heterogeneity in cell populations derived from blood and tissues of lupus mouse models and human lupus patients (38–41). Here we report the first study that employs the unique advantages of single-cell sequencing technology to demystify the heterogeneity in Breg subsets present in lupus-prone mice before disease onset and in the context of active autoimmune disease.

Overall, scRNA-seq revealed that IL-10 producing B cells generated from lupus-prone mice at the active-disease stage exhibited unique spatial transcriptomic profiles as compared to cells from mice before disease onset that reflected a heightened pro-inflammatory environment and evidence of increased activation of cell stress response pathways. The most striking differences we found in the distribution of Breg subsets from active-disease mice were decreased representation of MZ B cell subsets and increased representation of PB-PC subsets as compared to pre-disease mice. MZ B cells are innate-like cells that typically retain poly-reactive/self-reactive B cell receptors (BCRs), rapidly produce natural antibodies, and participate in clearance of pathogens and cellular debris (42). Exposure to apoptotic cells can induce MZ B cells to secrete IL-10 and take on regulatory functions, such that IL-10 producing T2-MZP and MZ B cells have been shown to have a protective role in murine models of collagen-induced arthritis (3, 5, 43). The increased proportion of Bregs of the MZ lineage generated from lupus-prone mice before disease onset raises a few interesting possibilities. Because of the innate-like nature of MZ B cells and their roles in the rapid immune response to pathogens, it is not surprising that MZ lineage cells would also be among the first Breg subsets to be induced in response to early autoimmune stimuli. Then, the decreased capacity to induce production of IL-10 producing MZ B cells as disease progresses could be caused by exposure to increasing inflammation that would promote egress of MZ-lineage Bregs to the periphery and/or the expansion of autoreactive PB-PC subsets in the spleen. Importantly, our observation that B cells from active-disease mice exhibited decreased numbers but not decreased IL-10 production by MZ B cell subsets as compared to pre-disease mice suggests that this subset may be particularly important to control autoimmune disease pathogenesis in this model.

B cells of the PB-PC lineage have also been attributed with regulatory function through production of IL-10 and IL-35 and suppression of pro-inflammatory and autoinflammatory responses (7, 44). Importantly, PB-PCs do not inherently produce IL-10 at steady state, but they may be induced to produce IL-10 by inflammatory environments present during infection or disease (45). It has also been noted that splenic B10 cells are capable of differentiation into antibody-secreting cells (ASCs) after *in vitro* or *in vivo* stimulation (46). Thus, in addition to their well-established contribution to lupus pathogenesis through the production of autoantibodies, PB-PCs also have the capacity to act as suppressors of autoimmunity although the origins and functions of IL-10 producing PB-PCs in SLE patients or lupus-prone mice have not been elucidated.

The MRL/*lpr* lupus-prone mouse strain is characterized by elevated lymphoproliferation, including the outgrowth of PB-PCs (47), and in line with this, we observed an increased percentage of IL-10 producing PB-PCs induced from B cells originating from the autoinflammatory environment present during active disease. However, evidence from our work and others would suggest that these Bregs are more inflammatory in nature and ineffective mediators of immunosuppression. Single-cell transcriptional analysis revealed that PB-PCs from active-disease mice exhibited increased expression of inflammatory pathway genes, which differed from IL-10 producing PB-PCs induced in a normal immune response to bacterial infection (33). We also found greater evidence of terminally differentiated, class-switched PC generation including the presence of a Breg cluster expressing GC B cell markers in active-disease but not pre-disease mice, a finding that supports the conclusion that a population of IL-10 producing PCs was increased within the Breg population in active disease. Even though the percentage of IL-10 producing PCs was increased in the Breg population, the fraction of total PCs that produced IL-10 was significantly decreased in active disease, consistent with the conclusion that the onset of autoimmunity in MRL/*lpr* mice was associated with an expansion of PCs but a decrease in differentiation toward the Breg phenotype in this population. This could contribute to an imbalance in the ratio of pro-inflammatory and regulatory influences and lead to the progression from the pre-disease to active-disease stage. Regarding the regulatory function of active-disease PB-PCs, we found no evidence for defective *Il10* transcription, but did observe decreased IL-10 MFI in PB-PCs from active-disease mice suggesting that they may transcribe *Il10* but not produce IL-10 protein (48). This could be because of defects in IL-10 translation, deficiencies in positive signals needed for IL-10 secretion, or improper negative regulation of IL-10. It is also possible that PB-PCs from active-disease mice are ineffective at immunosuppression because of improper localization and, thus, not positioned to interact with and suppress autoreactive T cells.

This study provides insights into differences in IL-10 producing B cells generated from lupus-prone mice before disease onset and in the context of active autoimmune inflammation. However, there are limitations to the study design that might narrow the interpretation of these results. Because of the low numbers of endogenous splenic Bregs, in particular in an environment with low inflammatory stimuli, we chose to boost the overall numbers of IL-10 producing cells for use in downstream analyses through *in vitro* stimulation. Thus, the populations of Bregs identified by single-cell sequencing are not a definitive survey of Breg development *in vivo*, but rather are indicative of the potential landscape of Breg subsets that may develop through the course of disease progression. In addition, due to our focus on changes to IL-10 producing B cells, we cannot identify potential relationships between changes to the composition of Bregs and changes to the total B cell population in the spleen that occur in the context of active disease. Therefore, this work represents compelling justification for future studies utilizing larger numbers of IL-10^+^ cells analyzed *ex vivo*. Furthermore, analysis of Bregs from lupus-prone mice could then serve as references for analogous Breg populations derived from human lupus patients.

The role of Bregs in the suppression of autoimmune inflammation and impaired functionality in SLE patients emphasizes the importance of improving our understanding of the heterogeneity of Breg subsets and how changes to Breg subset composition and function could impact lupus pathogenesis. We have utilized scRNA-seq to transcriptionally profile Bregs present at different disease stages in lupus-prone MRL/*lpr* mice and identified Breg subsets associated with increased inflammatory gene signatures specific to mice with active disease. Future studies are needed to investigate the functional competence and localization of IL-10^+^ Bregs, and in particular Bregs from the MZ B cell and PB-PC lineages, from pre-disease and active-disease mice to further elucidate their contributions to disease onset and progression. This work may better inform investigation of Breg subsets and functional capacity in human lupus patients and support the development of therapies targeting deficiencies in Breg-mediated regulation to improve clinical outcomes of autoimmune pathology.

## Data availability statement

The data presented in the study are deposited in the NCBI GEO repository, accession numbers GSE242200 and GSE103458.

## Ethics statement

The animal study was approved by Virginia Tech Institutional Animal Care and Use Committee. The study was conducted in accordance with the local legislation and institutional requirements.

## Author contributions

AD: Conceptualization, Formal Analysis, Investigation, Methodology, Software, Visualization, Writing – original draft, Writing – review & editing. RA: Conceptualization, Formal Analysis, Investigation, Methodology, Visualization, Writing – review & editing. AG: Funding acquisition, Project administration, Supervision, Writing – review & editing. XL: Conceptualization, Funding acquisition, Methodology, Project administration, Resources, Supervision, Writing – review & editing. PL: Conceptualization, Funding acquisition, Methodology, Project administration, Resources, Supervision, Writing – review & editing.
