# Supplementary material for: Single-cell RNA sequencing analysis reveals the heterogeneity of IL-10 producing regulatory B cells in lupus-prone mice
Source: Front Immunol. 2023 Dec 14;14:1282770. doi: 10.3389/fimmu.2023.1282770 (PMC10752970; doi:10.3389/fimmu.2023.1282770)
Supplement: Supplementary file 1 [file DataSheet_1.docx]

Supplementary Material

**Single-cell RNA sequencing analysis reveals the heterogeneity of IL-10 producing regulatory B cells in lupus-prone mice**

**Andrea R. Daamen^1#^, Razan M. Alajoleen^2#^, Amrie C. Grammer^1^, Xin M. Luo^2*^, Peter E. Lipsky^1*^**

*** Correspondence:**

Peter E. Lipsky

peterlipsky@comcast.net

Xin M. Luo

xinluo@vt.edu


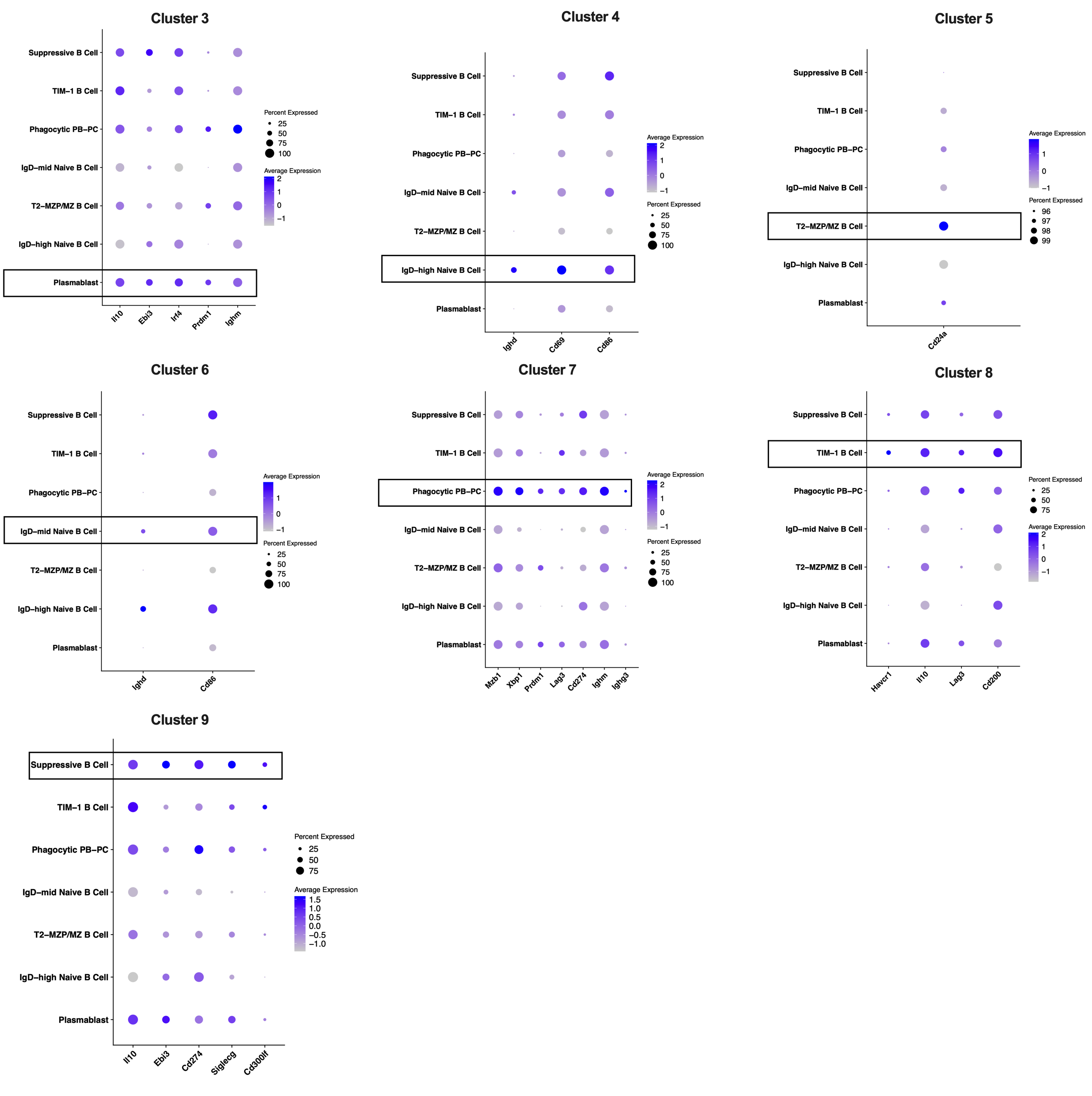


**Supplementary Figure 1.** **Pre-disease IL-10^+^ Breg annotated cluster markers**. Dot plots depicting selected markers used to annotate Bregs from lupus-prone mice at the pre-disease stage in Figure 2C. The annotation corresponding to each cluster number is indicated by a black rectangle. Dot size indicates percentage of cells in each cluster expressing each marker and dot color indicates average marker gene expression across the cluster.


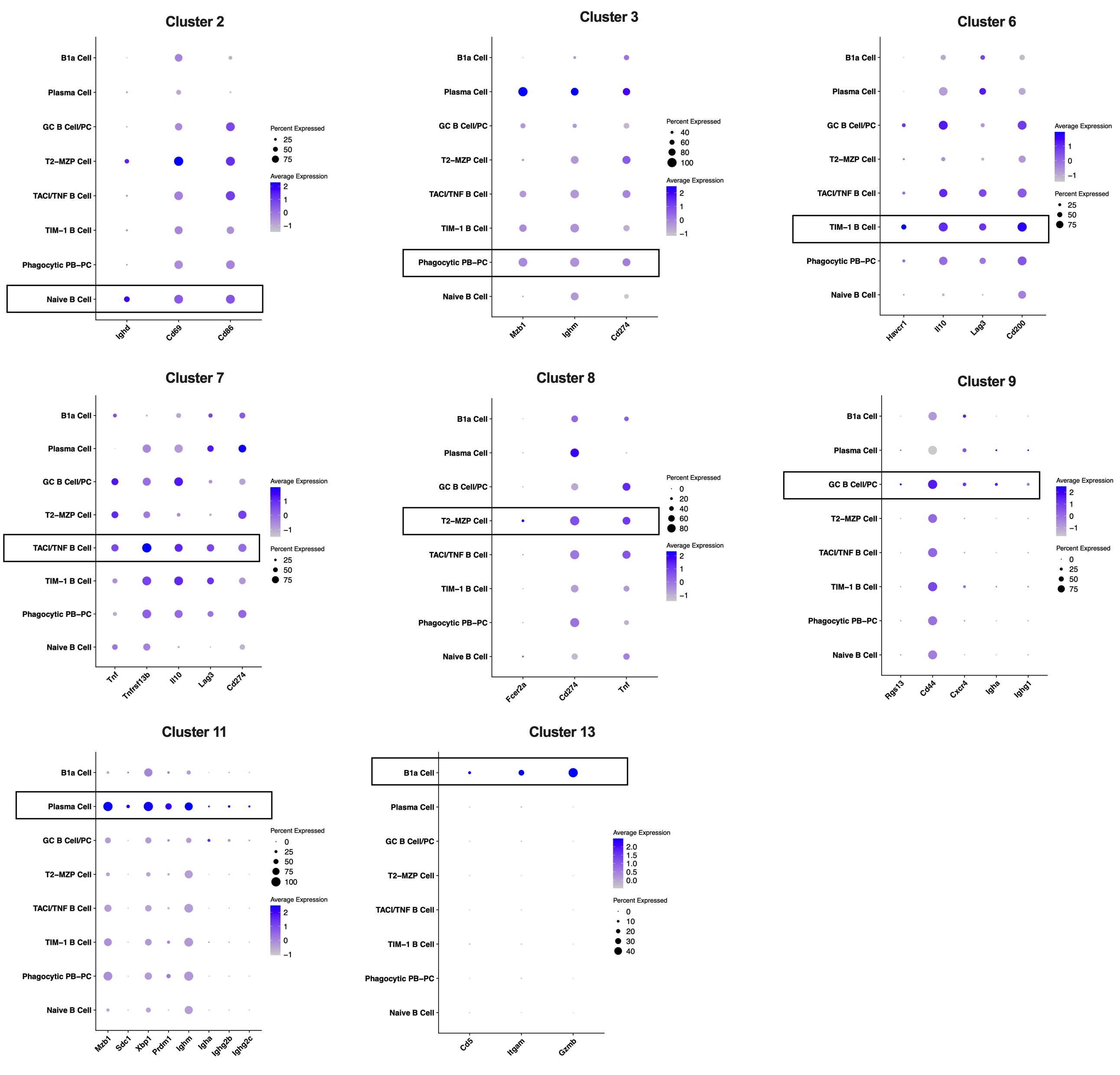


**Supplementary Figure 2.** **Active-disease IL-10^+^ Breg annotated cluster markers**. Dot plots depicting selected markers used to annotate Bregs from lupus-prone mice at the active-disease stage in Figure 3C. The annotation corresponding to each cluster number is indicated by a black rectangle. Dot size indicates percentage of cells in each cluster expressing each marker and dot color indicates average marker gene expression across the cluster.


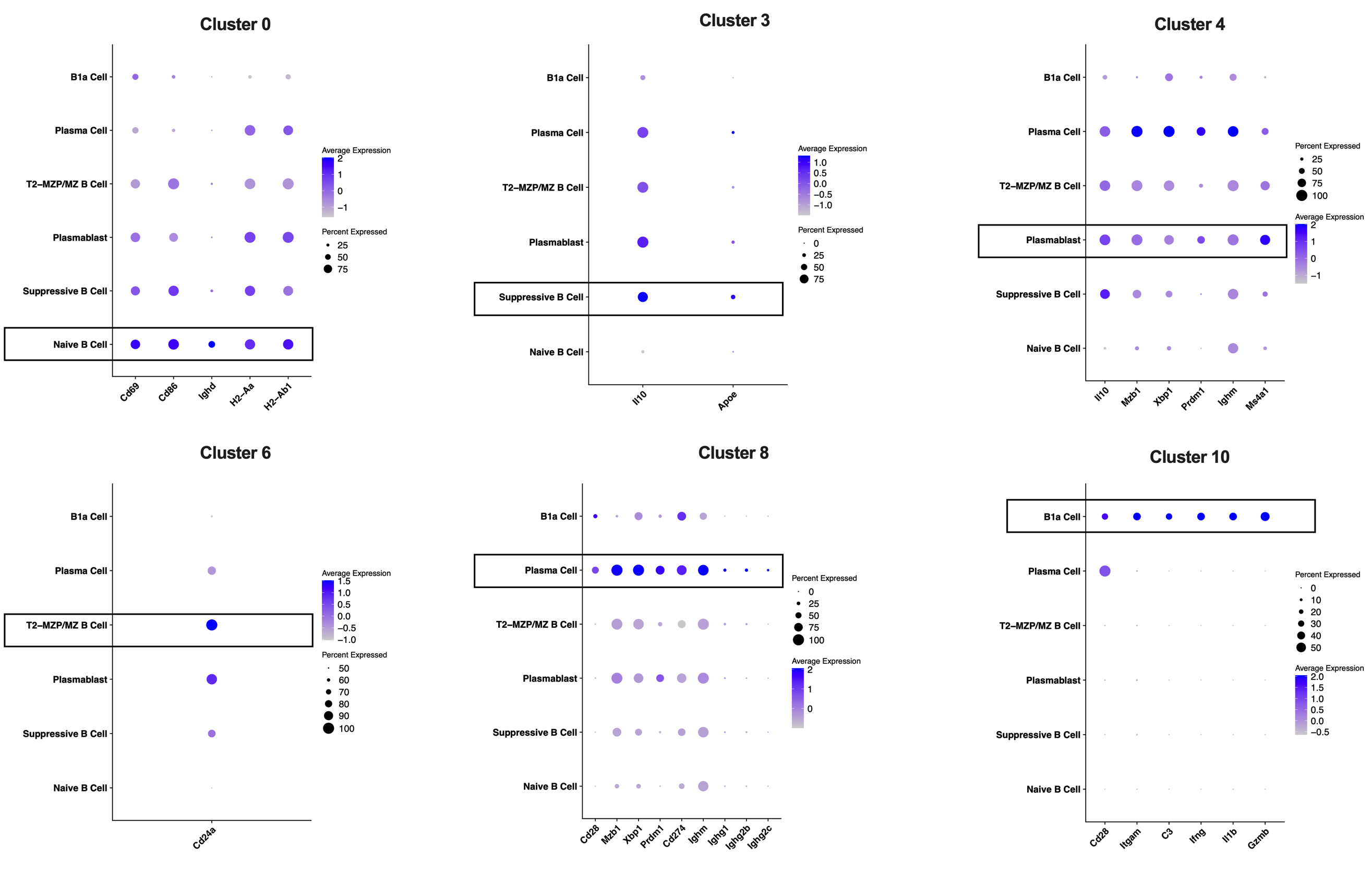


**Supplementary Figure 3.** **Integrated** **pre-disease IL-10^+^ Breg annotated cluster markers**. Dot plots depicting selected markers used to annotate Bregs from lupus-prone mice at the pre-disease stage in Figure 5C. The annotation corresponding to each cluster number is indicated by a black rectangle. Dot size indicates percentage of cells in each cluster expressing each marker and dot color indicates average marker gene expression across the cluster.


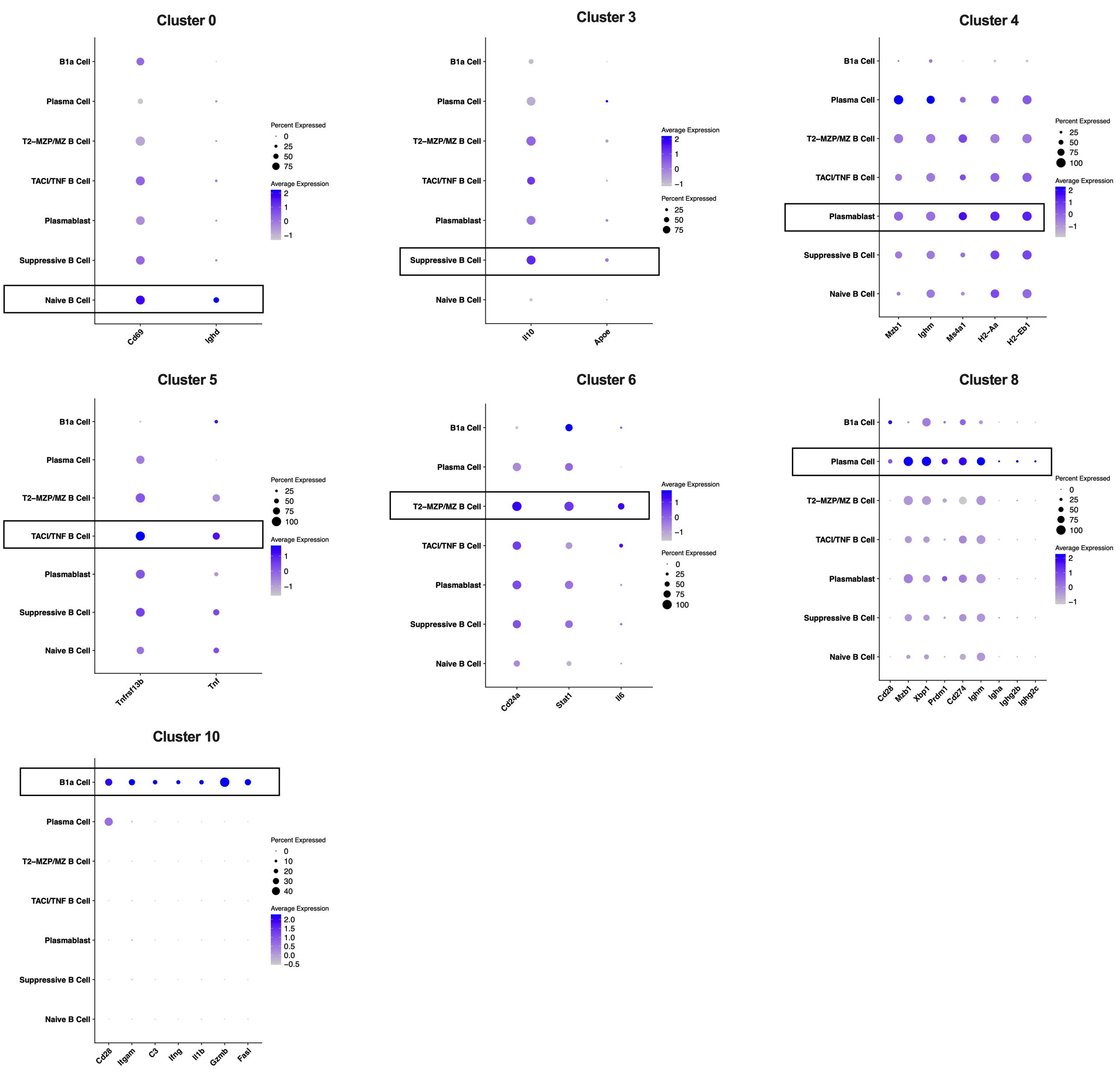


**Supplementary Figure 4.** **Integrated active-disease IL-10^+^ Breg annotated cluster markers**. Dot plots depicting selected markers used to annotate Bregs from lupus-prone mice at the active-disease stage in Figure 5E. The annotation corresponding to each cluster number is indicated by a black rectangle. Dot size indicates percentage of cells in each cluster expressing each marker and dot color indicates average marker gene expression across the cluster.


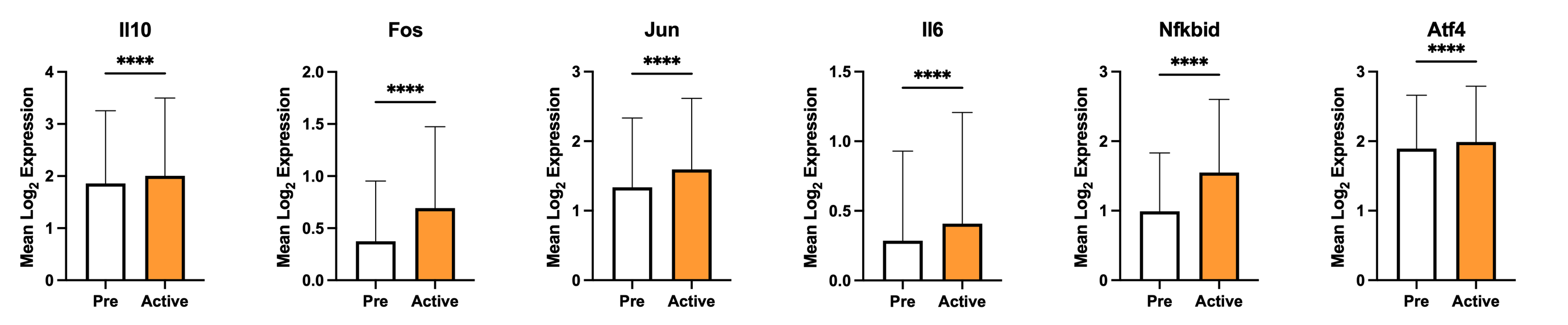


**Supplementary Figure 5.** **Differential expression of IL-10 pathway genes in active-disease mice**. Mean log_2_ expression values of genes in the IPA IL-10 Signaling Pathway canonical pathway signature between single-cells isolated from pre-disease and active-disease mice. **** *p* < 0.0001


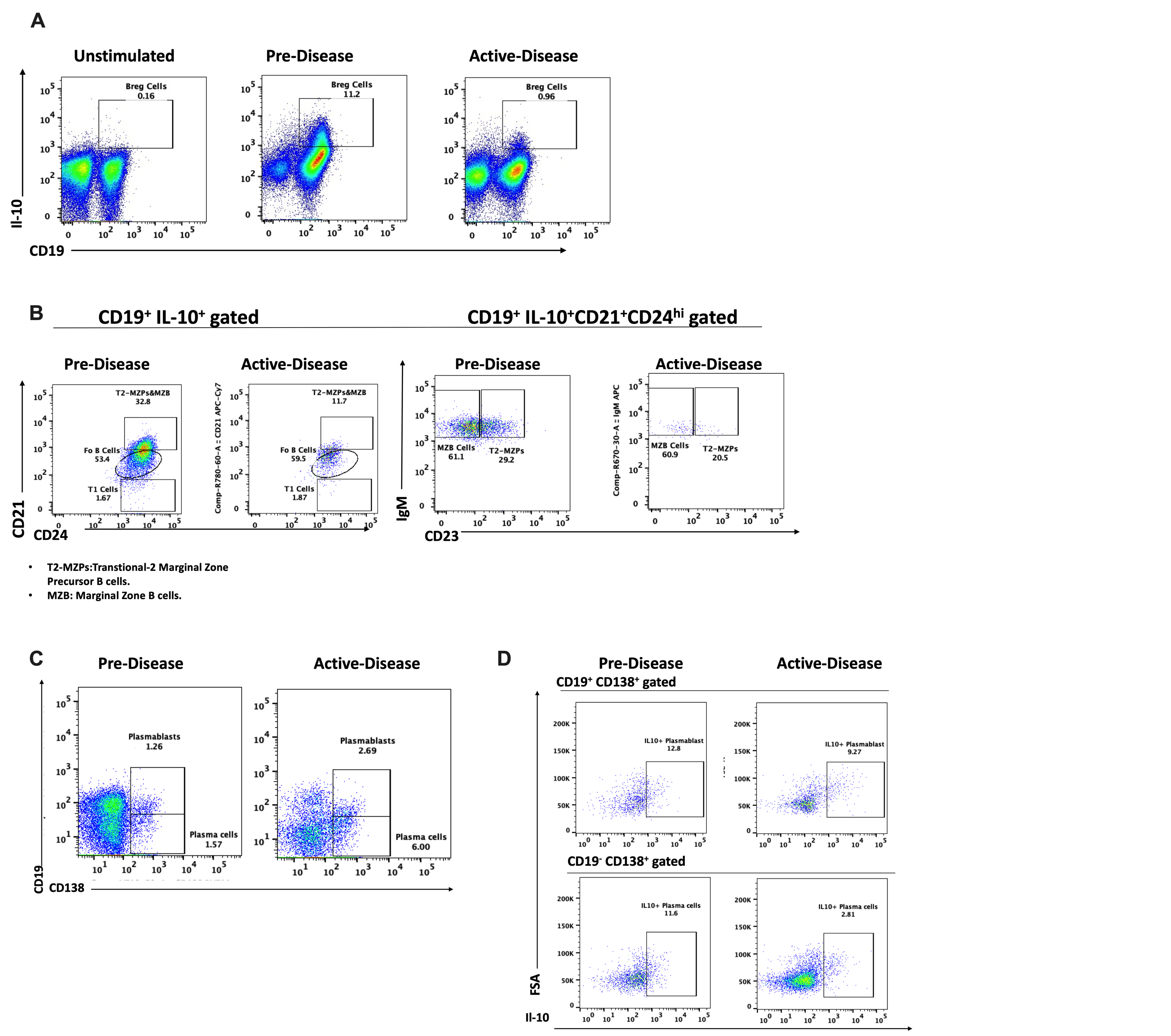


**Supplementary Figure 6.** **Flow cytometry gating strategy for IL-10^+^ Breg subsets.** Representative flow cytometry plots depicting the gating strategy for total splenic Bregs (**A**), T2-MZP/MZ B Cells , PB/PCs (**C**), and IL-10^+^ PB/PCs (**D**).
